# Changes in Coronal Alignment of the Knee Joint after Supramalleolar Osteotomy

**DOI:** 10.1155/2021/6664279

**Published:** 2021-02-19

**Authors:** Dong-Il Chun, Jahyung Kim, Sung Hun Won, Jaeho Cho, Jeongku Ha, Minkyu Kil, Young Yi

**Affiliations:** ^1^Department of Orthopedic Surgery, Foot and Ankle Center, Soonchunhyang University Seoul Hospital, 59, Daesagwan-ro, Yongsan-gu, Seoul, Republic of Korea; ^2^Department of Orthopedic Surgery, Soonchunhyang University Seoul Hospital, 59, Daesagwan-ro, Yongsan-gu, Seoul, Republic of Korea; ^3^Department of Orthopaedic Surgery, Chuncheon Sacred Heart Hospital, Hallym University, 77, Sakju-ro, Chuncheon-si, Gangwon-do, Republic of Korea; ^4^Department of Orthopaedic Surgery, Sports Medical Center and Sports Medical Research Institute, Inje University Seoul Paik Hospital, 85, 2-ga, Jeo-dong, Jung-gu, Seoul, Republic of Korea; ^5^Department of Orthopaedic Surgery, Seoul Foot and Ankle Center, Inje University Seoul Paik Hospital, 85, 2-ga, Jeo-dong, Jung-gu, Seoul, Republic of Korea

## Abstract

**Background:**

Assessing knee joint orientation changes after SMO may help clinical advancement in managing patients with ipsilateral ankle and knee joint arthritis. However, knee joint changes after supramalleolar osteotomy (SMO) have not been reported. We investigated changes in coronal alignment of the knee joint after SMO.

**Methods:**

In this multicentre study, from January 2014 to December 2018, 47 ankles with varus osteoarthritis treated with SMO were retrospectively identified. Ankle joint changes were assessed using the tibiotalar angle, talar tilt angle, and lateral distal tibial angle (LDTA); knee joint changes using the medial proximal tibial angle (MPTA), medial and lateral joint space widths (mJSW and lJSW, respectively), and medial and lateral joint line convergence angles (JLCA); and lower limb alignment changes using mechanical axis deviation angle (MADA) and the hip-knee-ankle (HKA) angle measured on full-length anteroposterior radiographs of the lower extremity. Correlation analysis and binary logistic regression analysis were performed.

**Results:**

Postoperatively, LDTA (*p* < 0.001) and tibiotalar angle (*p* < 0.001) significantly changed, indicating meaningful improvement in the ankle joint varus deformity. Regarding the knee joint changes, JLCA significantly changed into valgus direction (*p* = 0.044). As for lower limb alignment changes, MADA significantly decreased (*p* < 0.001), whereas the HKA angle significantly increased (*p* < 0.001). In univariate and multivariate logistic regression analyses, changes in the MADA (*p* < 0.001) and the HKA angle (*p* < 0.001) were significantly correlated with the correction angle.

**Conclusions:**

SMO remarkably improves ankle joint varus deformity, followed by significant lower limb alignment changes. Despite meaningful changes in JLCA, the relationship between the amount of osteotomy near the ankle joint and improvement in knee joint radiographic parameters was not significant. Radiographic parameters of the knee joint would less likely be changed following SMO.

## 1. Introduction

Supramalleolar osteotomy (SMO) of the ankle has been suggested to be a suitable treatment modality for varus ankle arthrosis by simultaneously redistributing the weight-bearing portion of the ankle joint and adjusting the varus alignment through lateral translation of the mechanical axis, followed by improved patient outcomes [1–4]. Similarly, high tibial osteotomy (HTO) is a useful treatment option to redistribute the knee joint load from areas of degeneration to intact articular surfaces in patients with varus knee alignment and medial compartment knee arthritis [5–7].

Although osteotomies are generally performed near the symptomatic joints, subsequent alignment changes can also influence the ipsilateral joints. Multiple studies have proven that varus ankle osteoarthritis in concurrence with ipsilateral varus knee osteoarthritis improved after HTO [4, 8, 9]. Takeuchi et al. [9] reported significant improvement in both clinical and radiologic outcomes of ankle osteoarthritis in patients who underwent HTO on the same limb. After osteotomy, reductions in both valgus inclination of the distal tibial joint surface and medial inclination of the talus were detected, followed by complete resolution of knee and ankle joint pain in all patients.

Similarly, the main goal of SMO is to realign the altered mechanical axis in order to normalize the joint loading within the ankle [10]. Haraguchi et al. [11] reported changes in the mechanical axis, including the hindfoot, among patients who underwent SMO, and Yi et al. [4] stated lateral deviation of the mechanical axis after SMO. Furthermore, Choi et al. [8] reported that the ankle joint orientation becomes parallel to the ground after HTO, and symptoms are affected by subsequent coronal alignment changes.

Consequently, defining changes in knee joint orientation after SMO may bring out significant clinical advancement in managing patients with ipsilateral ankle and knee joint arthritis, such as setting the order of priority between HTO and SMO or choosing adequate treatment modality to cover both joints. However, to our knowledge, no study has focused on knee joint changes after SMO. Therefore, we hypothesised that SMO would influence the alignment and function of the knee joint and such a change may affect knee joint orientation. This study is aimed at investigating changes in coronal alignment of the knee joint after SMO.

## 2. Material and Methods

This study was approved by the Institutional Review Board at our institution.

### 2.1. Patient Selection

**Figure 1:**
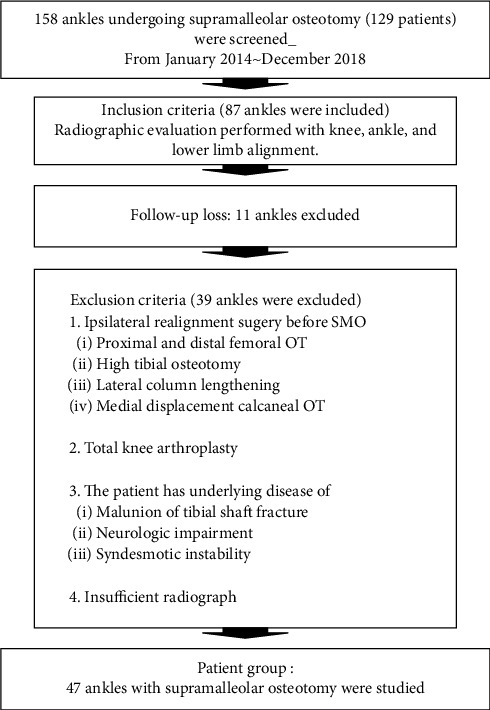
Flowchart showing the inclusion and exclusion criteria for the patients in this study.

This is a multicentre study performed by three surgeons at three different institutions under the same operation technique and rehabilitation program. From January 2014 to December 2018, 158 consecutive ankles (129 patients) with varus osteoarthritis treated with SMO were retrospectively identified. The indications for SMO included symptomatic Takakura IIIa varus ankle arthritis characterised with medial gutter narrowing and varus lower limb alignment. Contraindications were Takakura IIIb varus ankle arthritis and end-stage ankle arthritis. Among these patients, 87 patients who were concurrent with a standing whole-leg anteroposterior radiograph including bilateral knee and ankle joints were evaluated. 11 patients were lost during follow-up, and 39 patients were excluded following the exclusion criteria. Finally, 47 ankles in 45 patients (17 men and 28 women) were chosen for the study (Figure 1). The mean age was 56.3 years (range, 44 to 75 years). Comparative analysis was performed using preoperative and postoperative radiographic measurements among all included patients.

### 2.2. Operative Procedure

A 7 cm longitudinal incision is made along the anteromedial aspect of the distal tibia. Two Kirschner wires are inserted about 5 cm above the ankle mortise, just proximal to the tibiofibular syndesmosis, under fluoroscopic image intensifier in order to guide the osteotomy. Then, a periosteal incision of less than 1 cm is made along the site to perform the osteotomy, in an effort to maintain as many soft tissue attached to the distal bone fragment as possible. The osteotomy is performed parallel to the ankle mortise, and the lateral cortex at the apex of the distal part of the tibia must be preserved so that it can be used as a hinge. After completing the osteotomy, the distal osteotomized fragment is shifted inferiorly by introducing an osteotome through the medial aspect of the osteotomized site. Intraoperative visualization and fluoroscopy are used to evaluate the adequacy of the correction angle and lower limb alignment. Then, the osteotomy is fixed with 7-hole dynamic compression plate, in order to place three holes proximal and three holes distal to the osteotomy site. The gap is filled with autologous or allogenic bone graft.

### 2.3. Radiological Evaluation

**Figure 2:**
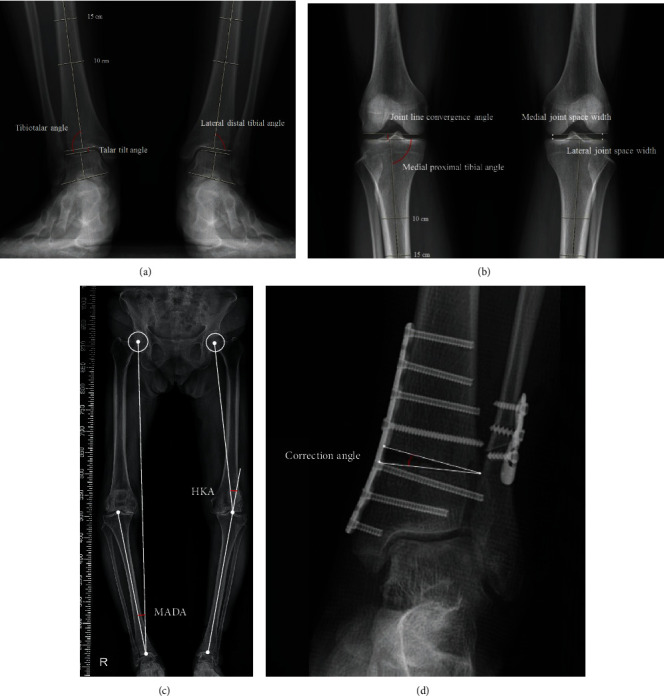
(a) Weight-bearing ankle anteroposterior view showing measurement of the lateral distal tibial angle (LDTA), tibiotalar angle (TTA), and talar tilt angle. The tibial axis was drawn by bisecting two pairs of points along the tibial shaft cortex, drawn 100 and 150 mm proximal to the tibial plafond. (b) Weight-bearing knee anteroposterior view showing measurement of the medial proximal tibial angle (MPTA), joint line convergence angle (JLCA), and medial and lateral joint space widths. The tibial axis was drawn by bisecting two pairs of points along the tibial shaft cortex, drawn 100 and 150 mm distal to the tibial plateau. (c) Full-length anteroposterior radiograph of the lower extremity showing measurement of the mechanical axis deviation angle (MADA) and hip-knee-ankle (HKA) angle. (d) Weight-bearing knee anteroposterior view showing correction angle after SMO.

The weight-bearing anteroposterior ankle, knee radiographs, and full-length anteroposterior radiographs were taken in all patients at preoperative and final follow-up period of at least 1 year after the surgery. In an effort to detect detailed changes in the radiographic parameters, measurements were performed on the tenfold magnified radiographs. Changes in the ankle joint were assessed with the use of the tibiotalar angle, talar tilt angle, and lateral distal tibial angle (LDTA) (Figure 2(a)) [8, 12]. Measurement was performed on weight-bearing anterior-posterior ankle radiograph taken with the patient standing, feet shoulder width apart, and second toes parallel to the coronal plane. The talar tilt angle was defined by the tibial and talar articular surfaces in the ankle joint. The tibiotalar angle was defined as the angle between the anatomical axis of the tibia and the line drawn parallel to the talar dome. The LDTA was defined as the angle between the tibial anatomical axis and the distal tibial articular surface on the standing ankle anteroposterior radiographs.

In addition, changes in the knee joint were assessed with medial proximal tibial angle (MPTA), medial and lateral joint space widths (mJSW and lJSW, respectively) [13], and medial and lateral joint line convergence angles (JLCA) [14]. Measurement was performed on weight-bearing anteroposterior knee radiographs taken with the patient standing, feet shoulder width apart, and both patella heading forward (Figure 2(b)). MPTA was defined by the angle between the tibial mechanical axis and the articular surface line of the proximal tibia. mJSW and lJSW were measured as the shortest distance between the distal femur and the proximal tibia for the medial and lateral joint spaces of each knee. JLCA was measured as the angle formed between a line tangential to the distal femoral condyle and the tibial plateau.

Moreover, changes in lower limb alignment were assessed with mechanical axis deviation angle (MADA) [12] and hip-knee-ankle (HKA) angle [8] measured on full-length anteroposterior radiograph of the lower extremity. MADA was defined as the angle formed by the line connecting the hip centre to the ankle centre and the tibial mechanical axis, while the HKA angle was the angle between the mechanical axes of the femur and the tibia (Figure 2(c)).

Lastly, the correction angle of SMO was measured as the angle between the proximal and distal osteotomized ends (Figure 2(d)).

### 2.4. Statistical Analysis

Patient demographics and preoperative and postoperative radiographic measurements are described as average value ± standard deviation. Although all the radiographic measurements were measured to three decimal points, the angle measurements were described to one decimal point. Changes in the radiographic parameters of the ankle, knee, and lower limb alignment before and after the surgery were analysed using paired *t*-test or signed rank test. Shapiro-Wilk normality test was performed on all measured data. Correlation analysis of the correction angle after SMO and preoperative and postoperative changes in the radiographic parameters was performed with Pearson correlation coefficient and Spearman's rank correlation coefficient. Binary logistic regression analysis with stepwise selection method was performed over values with significant correlation. Statistical analyses were performed using SPSS version 20.0 (IBM Corp., Armonk, NY, USA), and *p* values < 0.05 were considered statistically significant.

## 3. Results

Interobserver and intraobserver reliability was determined by calculating the interclass correlation coefficients for continuous data, and the reliability of all radiographic parameters was above 0.8 (Table 1). The target correction angle of the SMO was to rectify LDTA into 87°, and the valgus angulation difference was an average of 7.3° ± 6.0°.

Preoperative and postoperative changes in radiographic parameters of the ankle were as follows. The LDTA significantly changed from 94.7° ± 4.1° to 87.5° ± 5.7° with an average of 7.2° ± 6.0° valgus (*p* < 0.001). The tibiotalar angle also significantly improved from 95.4° ± 3.6° to 89.5° (87.4°, 93.4°) with 4.1° (-9.1°, -0.4°) valgus (*p* < 0.001). The talar tilt angle changed from an average of 2° (1.1°, 3.2°) to 1.8° (1.1°, 2.9°) with no significant difference.

**Figure 3:**
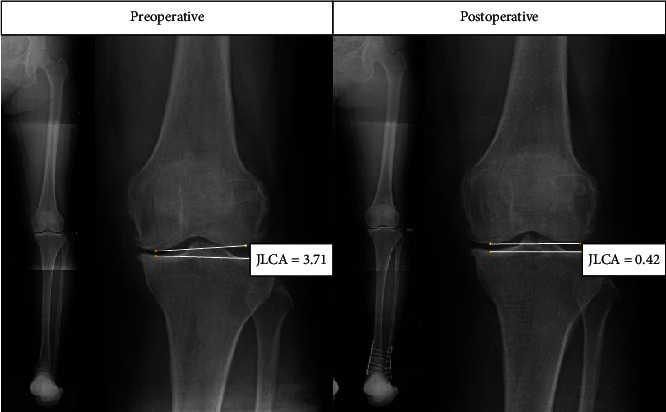
Example of a 57-year-old woman who underwent supramalleolar osteotomy. Preoperative and postoperative anteroposterior radiographs showing the JLCA changed from 3.71° to 0.42° after surgery (JLCA = joint line congruence angle).

Changes in the radiographic parameters of the knee joint were as follows. The MPTA changed from an average of 88.1° (87.0°, 89.3°) preoperatively to 88.3° (87.1°, 90.0°) postoperatively, and no significant difference was found. No significant changes were found in mJSW and lJSW from 5.15 ± 1.13 mm and 5.77 ± 1.08 mm to 5.32 ± 1.22 mm and 6.02 ± 1.51 mm, respectively. However, the JLCA changed from 1.5° (0.8°, 2.1°) to 1.9° ± 1.3°, showing 0.2° (-0.2°, 0.4°) valgisation (*p* = 0.044) (Figure 3).

As for radiographic changes in the lower limb alignment, MADA significantly decreased from 2.6° ± 1.3° preoperatively to 2.0° ± 1.5° postoperatively (*p* < 0.001), while the HKA angle significantly increased from 176.0° ± 2.0° preoperatively to 176.8° (175.7°, 177.8°) postoperatively (*p* < 0.001) (Table 2). In terms of correlation between the correction angle and radiologic parameters, the HKA angle (*r* = 0.440, *p* < 0.001) and MADA (*r* = −0.580, *p* < 0.001) differences in mechanical axis were correlated (Table 3).

Among significant variables, in both univariate and multivariate logistic regression analyses using a stepwise selection method, changes in MADA and HKA angle significantly correlated with the correction angle (Table 4).

## 4. Discussion

To the best of our knowledge, this is the first study that focused on coronal changes in the knee joint after ankle joint SMO. Although changes in the tibiotalar or subtalar joint after HTO have been described in previous studies, no study stated changes in the knee joint after SMO. Recently, treatment option toward homonymous varus knee and ankle osteoarthritis has been reported in the literature [9]. Although it is commonly recommended to approach the proximal degenerative joint ahead, defining changes in the untouched joint induced by the operated joint could be an important issue because the two joints are in line with each other based on the mechanical axis.

In this study, changes in the knee joint and lower limb alignments were measured in addition to those in the ankle joint, and significant correlation was found between the correction angle and limb alignment. That is, as the correction angle increased after SMO, mechanical deviation decreased and the HKA angle changed. However, when JLCA was excluded, no significant correlations were found among other radiographic parameters of the knee joint.

Several studies have reported the correlation between the HTO and coronal lower limb alignment [15–17]. Lee et al. [16] compared the JLCA among patients divided into three different groups after HTO: undercorrection (weight-bearing line (WBL) ratio, <57%), overcorrection (WBL ratio, >67%), and acceptable correction (WBL ratio, 57–67%) groups. The JLCA was measured before and after surgery and compared between the three groups. Difference between the pre­ and postoperative values of the JLCA showed a stronger correlation than those of the MAD. Such differences in the JLCA after HTO are related to an increase in the knee medial joint width and a decrease in the lateral joint width. Changes in MPTA were also found in the case of open-wedge HTO because of alteration in the morphology of the proximal tibia.

In this study, a significant change in the JLCA was detected after SMO, probably because of the fact that the knee joint becomes affected by the valgus force after medial open-wedge osteotomy performed at its distal level [18]. However, although statistically significant, such change was as minimal as 0.2 degrees and was not significantly correlated with the correction angle in the regression analysis. Similarly, no significant change was detected on the MPTA or mJSW and lJSW after surgery, while these factors were not related to the correction angle in the regression analysis.

**Figure 4:**
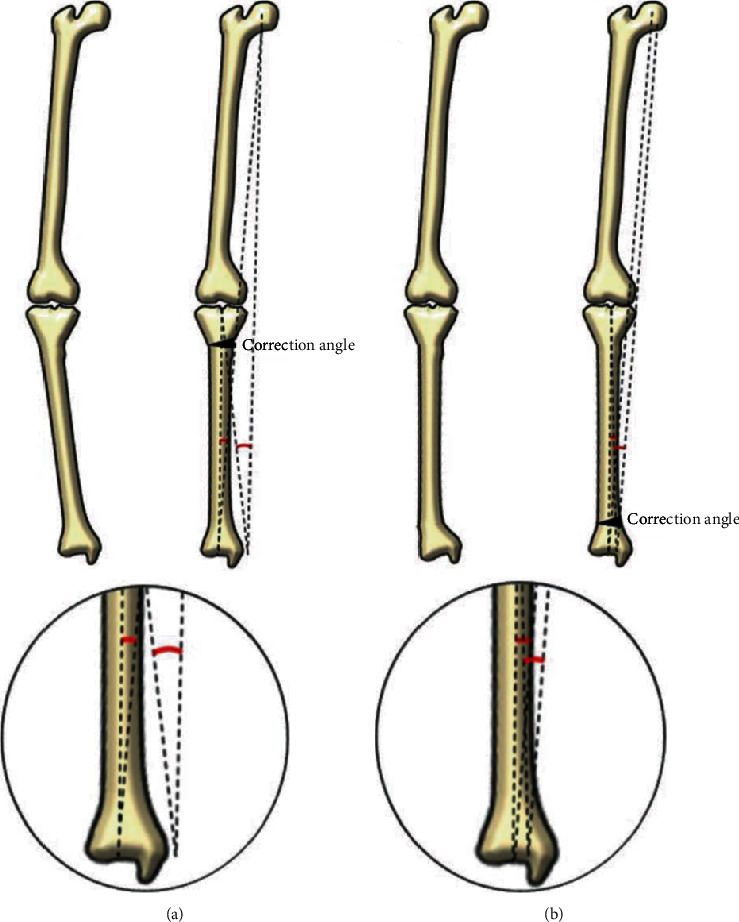
Schematic illustration of alterations in lower limb alignment. (a) Changes in the mechanical axis deviation after high tibial osteotomy on the lower limb alignment. (b) Changes in the mechanical axis deviation after supramalleolar osteotomy on the lower limb alignment (the same amount of correction angle was made in both surgeries).

Variety of explanations could be made concerning such insignificant changes in the knee joint following SMO. First, as osteotomy is performed on the distal portion of the tibia in SMO, no direct structural change around the knee joint can be achieved. Although changes in the JLCA formed by the distal femur and proximal tibia were found, operative correction cannot be performed either. Second, the knee joint becomes less corrected after SMO because the more distal the osteotomy is performed, that is, at the level of the metaphysis of distal tibia, the lesser the mechanical axis changes. It is depicted in Figure 4 showing inferior alteration in mechanical axis in SMO compared with HTO, even with the same correction angle (Figure 4). Third, the knee joint is surrounded by relatively abundant soft tissue including medial collateral ligament, articular cartilage, or meniscus. Owing to the untouched, normal integrity of these structures acting as restraints to valgus forces around the knee joint, change in the mechanical axis caused by SMO, which becomes larger with the amount of correction angle, could eventually be counterbalanced [19]. Lastly, other factors including patients' weight or hindfoot compensation might have buffered the substantial changes in the knee joint [15, 17].

Another possible explanation would be the difference in the moment arms between two surgeries. Many studies have reported that the ankle joint has a longer moment arm than the knee joint when HTO is performed [8, 20]. Therefore, changes could be more prominent in the ankle joint than in the knee joint. However, in the case of SMO, given the shorter moment arm than the ankle joint, the influence of osteotomy would be inferior in the knee joint.

This study has some limitations. First, patients with relatively early stage of knee osteoarthritis were included, as no operation was performed on the affected knee at the time of SMO. For this reason, direct influence of SMO on the advanced knee osteoarthritis could not be analysed. However, as changes in lower limb alignment had minimal effect on the knee joint, insignificant change in advanced arthritic knee joint could be assumed in the same manner. Second, external factors such as administration of painkillers or prolonged period of nonweight bearing related to cast application could not be controlled after surgery. Nevertheless, as partial weight bearing was allowed in 4 weeks, full weight bearing in 8 weeks, and an attempt to return to the normal activity initiated within 3 months after surgery, we thought that the follow-up period of 1 year would be long enough to neutralise the non-weight-bearing period. Third, clinical outcomes were not evaluated simultaneously. Although no significant correlation was found between the correction angle and radiographic parameters of the knee joint, changes in lower limb alignment might positively influence the clinical symptoms. However, as additional pain control using medications was performed during the 1-year follow-up period, we thought it would be awkward to establish a clear relationship between clinical outcomes and radiographic parameters. As a result, we thought that radiographic analysis would be sufficiently meaningful. Fourth, this is a retrospective study with a relatively small sample size of 47 cases. When we performed a post hoc power analysis upon all the variables in the final regression model, it turned out that more sample size would be needed in order to verify the HKA angle in the succeeding study. Last, as this study solely focused on radiographic parameters in the coronal plane, the possible influence of changes in the other planes could have been neglected. In addition, the relationship between changes in the subtalar joint and knee joint could not be evaluated. These factors would have to be additionally considered in the future study.

In this study, our hypothesis was dismissed, as no significant change was found in the knee joint after SMO. As mechanical axis transition in association with the amount of correction angle was detected, a substantial amount of osteotomy near the ankle joint has to be performed to sufficiently rotate the mechanical axis to affect both the ankle and knee joints. Nevertheless, correction through osteotomy without limit is impossible to perform, and both functional maintenance and bony union of the osteotomy site have to be taken into account.

## 5. Conclusion

SMO can lead to a remarkable improvement in the ankle joint varus deformity, followed by significant changes in lower limb alignment. Although meaningful changes were also detected in terms of JLCA, the relationship between the amount of osteotomy near the ankle joint and improvement in radiographic parameters of the knee joint was not significant. Therefore, radiographic parameters of the knee joint would less likely be changed following SMO.

## Figures and Tables

**Table 1:** Inter- and intraobserver reliability of the radiologic parameters.

Correction angle	Interobserver reliability	Intraobserver reliability
Ankle		
Tibiotalar angle	0.94 (0.87 to 0.99)	0.93 (0.86 to 0.99)
Talar tilt angle	0.91 (0.81 to 0.97)	0.91 (0.79 to 0.97)
LDTA	0.92 (0.87 to 0.97)	0.91 (0.79 to 0.97)
Knee		
MPTA	0.92 (0.87 to 0.97)	0.94 (0.87 to 0.99)
mJSW	0.95 (0.87 to 0.99)	0.92 (0.87 to 0.97)
lJSW	0.95 (0.88 to 0.99)	0.93 (0.86 to 0.99)
JLCA	0.93 (0.86 to 0.99)	0.90 (0.79 to 0.97)
Mechanical axis		
HKA angle	0.90 (0.79 to 0.97)	0.91 (0.79 to 0.97)
MADA	0.88 (0.77 to 0.96)	0.89 (0.76 to 0.98)

HKA: hip-knee-ankle; JLCA: joint line convergence angle; lJSW: lateral joint space widths; MADA: mechanical axis deviation angle; mJSW: medial joint space widths; MPTA: medial proximal tibial angle.

**Table 2:** Changes and significance in radiologic parameters of pre- and postoperative ankle, knee, and lower limb alignment.

	Preoperative	Postoperative	Change (post-pre)	*p* value
	Mean ± SD or median (IQR)			
Ankle				
LDTA	94.7 ± 4.1	87.5 ± 5.7	−7.2 ± 6.0	***<0.001***
Talar tilt angle	2 (1.1, 3.2)	1.8 (1.1, 2.9)	-0.6 (-1.4, 0.6)	0.1214
Tibiotalar angle	95.4 ± 3.6	89.5 (87.4, 93.4)	-4.1 (-9.1, -0.4)	***<0.001***
Knee				
MPTA	88.1 (87.0, 89.3)	88.85 (87.1, 89.5)	0 (-0.2, 0.5)	0.3542
mJSW	5.15 ± 1.13	5.32 ± 1.22	0.17 ± 0.74	0.1203
lJSW	5.77 ± 1.08	6.02 ± 1.51	0.06 (-0.59, 0.78)	0.2665
JLCA	1.5 (0.8, 2.1)	1.9 ± 1.3	0.2 (-0.2, 0.4)	***0.0444***
Mechanical axis				
HKA angle	176.0 ± 2.0	176.8 (175.7, 177.8)	0.5 (0.2, 1.3)	***<0.001***
MADA	2.6 ± 1.3	2.0 ± 1.5	-0.7 (-1, -0.1)	***<0.001***

IQR: interquartile range; HKA: hip-knee-ankle; JLCA: joint line convergence angle; lJSW: lateral joint space widths; MADA: mechanical axis deviation; mJSW: medial joint space widths; MPTA: medial proximal tibial angle.

**Table 3:** Correlation between the correction angle and radiologic parameters.

	Correlation with correction angle	
	*r*	*p* value
Knee		
MPTA difference	-0.080	0.587
mJSW difference	0.080	0.590
lJSW difference	0.235	0.108
JLCA difference	0.115	0.436
Mechanical axis		
HKA angle difference	0.440	<0.001
MADA difference	-0.580	<0.001

HKA: hip-knee-ankle; JLCA: joint line convergence angle; lJSW: lateral joint space widths; MADA: mechanical axis deviation angle; mJSW: medial joint space widths; MPTA: medial proximal tibial angle.

**Table 4:** Binary regression analysis of the radiologic parameters.

	Univarable analysis				Multivariable analysis			
	Beta	95% CI		*p* value	Beta	95% CI		*p* value
		Lower	Upper			Lower	Upper	
*Knee*								
MPTA	-0.128	-0.460	0.204	0.441				
mJSW	0.198	-0.536	0.931	0.590				
lJSW	0.162	-0.210	0.534	0.385				
JLCA	-0.059	-0.397	0.278	0.726				
*Mechanical axis*								
HKA angle	0.001	-0.335	0.336	0.996	-0.301	-0.614	0.012	0.049
MADA	-0.94	-1.421	-0.459	<0.001	-1.157	-1.676	-0.638	<0.001

HKA: hip-knee-ankle; JLCA: joint line convergence angle; lJSW: lateral joint space widths; MADA: mechanical axis deviation angle; mJSW: medial joint space widths; MPTA: medial proximal tibial angle.

## Data Availability

These data were available to us as staffs of three different institutions including Seoul Paik Hospital of Inje University, Soonchunhyang Seoul Hospital, and Chuncheon Sacred Heart Hospital Hallym University. These data are protected by the Ministry of Health and Welfare and patient privacy laws in Korea; no public links are available to these protected health information datasets. These data will be made available to others after appropriate data privacy and human subject approvals needed by the institution. Requests should be sent to 20vvin@naver.com.
